# Antibiotic Susceptibility Patterns of *Aggregatibacter actinomycetemcomitans* and *Porphyromonas gingivalis* Strains from Different Decades

**DOI:** 10.3390/antibiotics8040253

**Published:** 2019-12-06

**Authors:** Eva M. Kulik, Thomas Thurnheer, Lamprini Karygianni, Clemens Walter, Anton Sculean, Sigrun Eick

**Affiliations:** 1 Department of Oral Health & Medicine, University Center for Dental Medicine, University of Basel, 4058 Basel, Switzerland; 2 Clinic of Conservative and Preventive Dentistry, Division of Oral Microbiology and Immunology, Center of Dental Medicine, University of Zurich, 8032 Zurich, Switzerland; thomas.thurnheer@zzm.uzh.ch (T.T.); lamprini.karygianni@zzm.uzh.ch (L.K.); 3 Department. of Periodontology, Endodontology and Cariology, University Center for Dental Medicine, University of Basel, 4058 Basel, Switzerland; clemens.walter@unibas.ch; 4 Department of Periodontology, School of Dental Medicine, University of Bern, 3001 Bern, Switzerland; anton.sculean@zmk.unibe.ch (A.S.); sigrun.eick@zmk.unibe.ch (S.E.)

**Keywords:** minimal inhibitory concentrations, periodontal pathogens, beta lactams, moxifloxacin

## Abstract

The aim of this study was to determine the antibiotic susceptibility patterns of 57 *Aggregatibacter actinomycetemcomitans* and 56 *Porphyromonas gingivalis* strains isolated from subgingival biofilm samples of periodontitis patients in Switzerland from 1980 to 2017. The minimal inhibitory concentrations (MIC) of the most commonly used antibiotics in periodontal therapy (amoxicillin, metronidazole, azithromycin, and doxycycline) or in severe body infections (amoxicillin/clavulanic acid, clindamycin, ertapenem, and moxifloxacin) were determined. Furthermore, all the strains were screened for beta-lactamase activity and the presence of selected resistance genes (*cfx*A, *erm*F, and *tet*Q). Overall, there was no significant increase in MIC values over the 37‑year period. Two of the most recent *P. gingivalis* isolates yielded the highest MIC values. The first isolate was *erm*F-positive with MIC values >8 µg/mL, 2 µg/mL, and 0.25 µg/mL for clindamycin, azithromycin, and moxifloxacin, respectively. The second isolate showed a high MIC value of 4 µg/mL for moxifloxacin, which was associated with a confirmed single-point mutation in the quinolone resistance-determining region (QRDR) of the *gyr*A gene. Although there was no significant increase in the antibiotic resistance among the oral bacterial isolates tested, the detection of resistant *P. gingivalis* isolates underlines the need to optimize the antibiotic therapeutic protocols in dentistry.

## 1. Introduction

Due to increased and often unrestricted consumption of antibiotics both in humans and in animal husbandry, the development of resistance against antibiotics has become a global healthcare threat leading to higher medical costs, prolonged hospital stays, and increased mortality [1,2]. There is an urgent need for change in the prescription and use of antibiotics [3,4]; however, so far, strategies based on antibiotic stewardships, infection prevention and control programs were unable to control the increase in antimicrobial resistance [5]. As there is also a lack of new antimicrobials that are effective against resistant bacteria, the knowledge of factors contributing to the spread of resistant microorganisms is of high importance [6].

A recent analysis in the U.S showed that dentists account for 13.2% of all antibiotic prescriptions among the antibiotic prescriptions providers, with periodontists having a higher prescription rate than general dentists [7]. Periodontal diseases are polymicrobial oral infections by predominantly Gram-negative capnophilic and anaerobic subgingival bacterial species such as *Aggregatibacter actinomycetemcomitans* and *Porphyromonas gingivalis* [8]. The virulence factors of *A. actinomycetemcomitans*, associated with severe periodontitis in young age, include leukotoxin, cytolethal distending toxin, and lipopolysaccharide [9]. *P. gingivalis* has high proteolytic activity and is considered a keystone pathogen in the initiation and progression of periodontal disease [10]. Besides the mechanical debridement of infected periodontal pockets, clinical treatment protocols for severe forms of periodontitis often involve the adjunctive use of antibiotics like amoxicillin, metronidazole, azithromycin, and tetracycline [11,12].

The objective of this study was to determine the antibiotic susceptibility among clinical isolates of *A. actinomycetemcomitans* and *P. gingivalis* isolated from Swiss periodontitis patients over four decades.

## 2. Results

In total, 57 *A. actinomycetemcomitans* and 56 *P. gingivalis* strains could be included in this study. Only one isolate per patient was considered in the analysis. As *P. gingivalis* strains were only available from 1990 on, isolates were not equally distributed over the different decades (Table 1).

Table 2 shows the MIC_50_ and MIC_90_ values for the tested antimicrobials. The results confirm the high resistance of *A. actinomycetemcomitans* against clindamycin (≥8 µg/mL) and metronidazole (≥32 µg/mL). The MIC_90_ values for ampicillin and amoxicillin/clavulanic acid were 2 µg/mL and 2/1 µg/mL, respectively, whereas the MIC_50_ and MIC_90_ values of the other antimicrobials were low. *P. gingivalis* yielded low MIC_50_ (0.0625–0.5 µg/mL) and MIC_90_ (0.125–2 µg/mL) values for all the antimicrobials tested.

**Figure 1:**
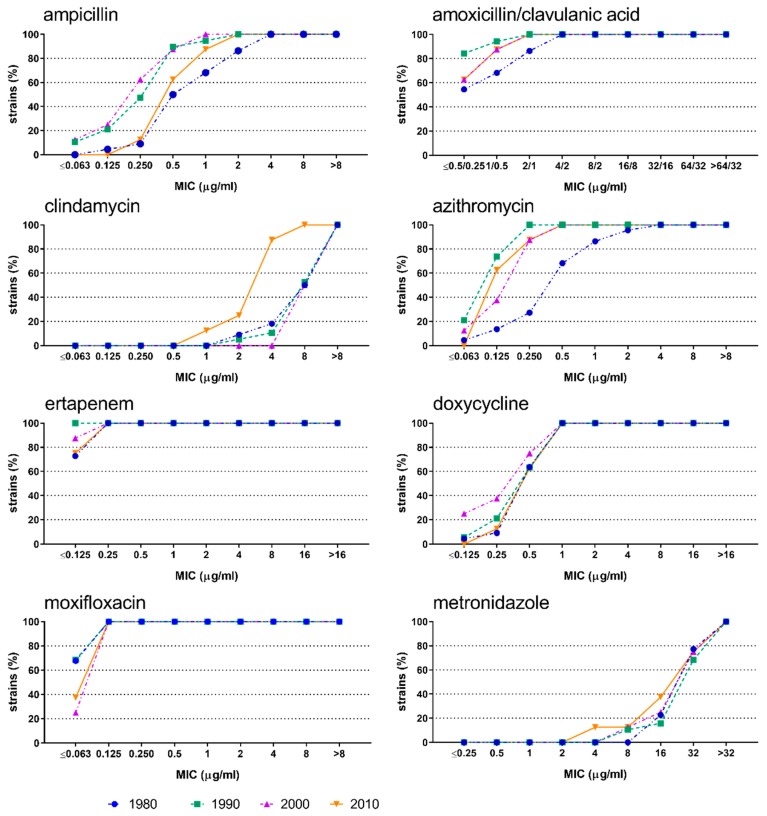
Cumulative minimal inhibitory concentrations against *Aggregatibacter actinomycetemcomitans* isolates. Results are shown for the antibiotics ampicillin, amoxicillin/clavulanic acid, clindamycin, azithromycin, ertapenem, doxycycline, moxifloxacin, and metronidazole during the decades 1980 (1980–1988; blue), 1990 (1990–1999; green), 2000 (2000–2009; purple), and 2010 (2010–2017; orange).

**Figure 2:**
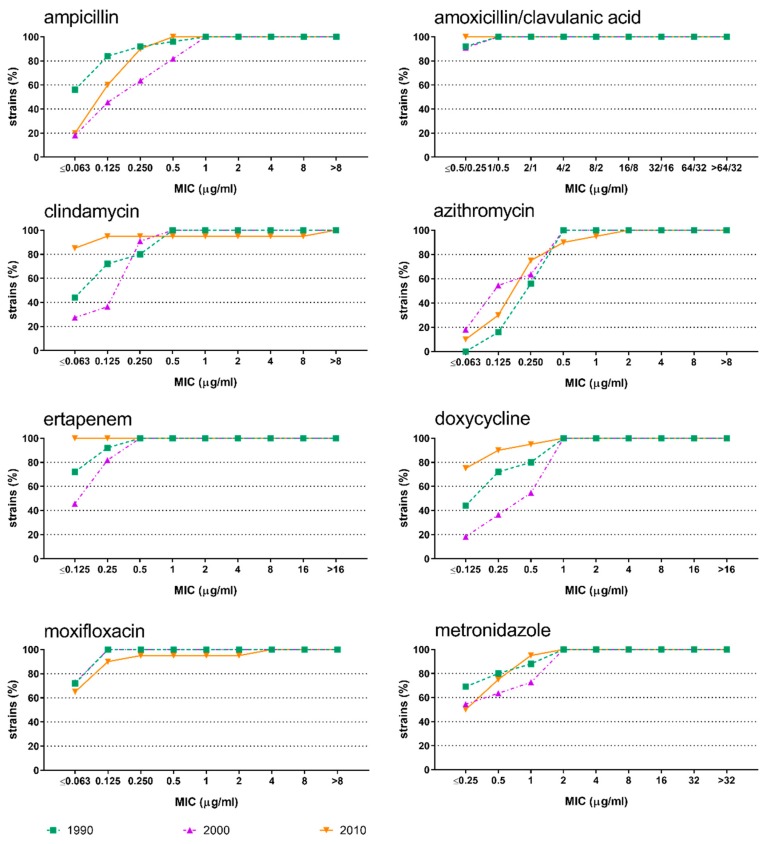
Cumulative minimal inhibitory concentrations against *Porphyromonas gingivalis* isolates. Results are shown for the antibiotics ampicillin, amoxicillin/clavulanic acid, clindamycin, azithromycin, ertapenem, doxycycline, moxifloxacin, and metronidazole during the decades 1990 (1990–1999; green), 2000 (2000–2009; purple), and 2010 (2010–2017; orange).

The susceptibility of *A. actinomycetemcomitans* (Figure 1) and *P. gingivalis* (Figure 2) to most of the antimicrobials did not change over time. *A. actinomycetemcomitans* showed statistically significant differences for azithromycin (*p* = 0.002) and clindamycin (*p* = 0.004). In particular, azithromycin exhibited the highest cumulative MIC values in the first decade (1980–1989), while clindamycin yielded the lowest cumulative MIC values in the last decade (2010–2017). Regarding *P. gingivalis*, statistically significant differences were observed for clindamycin (*p* = 0.006), ertapenem (*p* = 0.014), and doxycycline (*p* = 0.040).

The *cfx*A gene was not detected in any of the tested *P. gingivalis* and *A. actinomycetemcomitans* strains and the nitrocefin test was negative for all tested isolates. The *tet*Q gene was identified in two *P. gingivalis* strains, one of which yielded a MIC of 1 µg/mL for doxycycline.

Two *P. gingivalis* strains isolated in the last decade (2010–2017) showed the highest MIC values. One isolate was *erm*F-positive and had MIC values higher than 8 µg/mL, 2 µg/mL, and 0.25 µg/mL for clindamycin, azithromycin, and moxifloxacin, respectively. The second isolate had a high MIC value of 4 µg/mL for moxifloxacin. Sequence analysis of the quinolone resistance-determining region (QRDR) of the *gyr*A gene confirmed a gene mutation, namely a Ser-83 ≥ Phe substitution [13].

## 3. Discussion

Overall, an increase of MIC values among the tested *A. actinomycetemcomitans* and *P. gingivalis* isolates over the 37-year period could not be observed. Although only a limited number of clinical isolates was analyzed in this study, the results are in agreement with a previous Swiss study, where no increase was detected in antibiotic-resistant oral bacteria after comparing *Prevotella intermedia* and *A. actinomycetemcomitans* strains isolated during 1991–1994 with isolates collected during 2001–2004 [14]. The absence of an increasing antibiotic resistance among the tested bacteria associated with periodontal disease over the last 37 years might be attributed to the restricted use of antibiotics in the treatment of severe forms of chronic periodontitis at the dental university clinics of Basel, Bern, and Zurich. A questionnaire among dentists in dental practices in Switzerland confirmed that the prescription of antibiotics in Switzerland is selective and cautious [15]. In contrast, many prescriptions in the United States are considered to involve ‘inappropriate’ use of antibiotics [16].

In regard to the resistance profiles of *A. actinomycetemcomitans* and *P. gingivalis*, our results are comparable to data from the Netherlands [17]. Similarly, a high sensitivity of *P. gingivalis* to antibiotics commonly used in dentistry is found in the United States and Norway, whereas a significant amount of *A. actinomycetemcomitans* strains is resistant to tetracycline [18,19]. Antibiotic resistances of other oral anaerobic bacteria are of increasing clinical importance as well. In particular, oral *Prevotella* spp. isolated from periodontal abscesses were resistant to metronidazole (9.5%), amoxicillin (30.9%), clindamycin (38.1%), and doxycycline (4.8%). Their increased resistance was often related to the presence of *nim*, *cfx*A, *erm*F, and *tet*Q genes [20]. In another study, oral isolates of *P. intermedia* and *Prevotella buccae* yielded high MIC values for doxycycline and tetracycline [21]. In our study, the *erm*F gene was identified in accordance with a phenotypical resistance to clindamycin only in one *P. gingivalis* isolate. The *tet*Q gene was present in only one of the two respective *P. gingivalis* isolates associated with a slightly elevated MIC value to doxycycline.

In subgingival plaque, bacteria are organized as a complex biofilm. In general, MIC values are higher for bacteria in biofilms than for planktonic cultures [22]. Various biofilm models exist for analyzing antimicrobial susceptibility and first attempts are being made to determine the antibiotic concentrations that can inhibit all microorganisms present in a polymicrobial sample [23,24].

One of the *P. gingivalis* strains from our study, a strain isolated from a patient with rheumatoid arthritis in the decade 2010–2017, showed an elevated MIC value for the fluoroquinolone, moxifloxacin, which was due to a mutation in the *gyr*A gene. No resistance to quinolones has been reported in clinical oral isolates of *P. gingivalis* so far. However, moxifloxacin resistance in *P. gingivalis* strains could be induced in vitro by exposing *P. gingivalis* strains to subinhibitory concentrations of fluoroquinolones. In this study, published in 2004, resistance was shown to be due to a Ser-83 ≥ Phe substitution in the *gyr*A gene of the QRDR [13]. At that time, the adjunctive use of moxifloxacin was promoted in periodontal therapy because of the antibiotic’s ability to penetrate into tissues and cells of anaerobic periodontal pathogens in both the planktonic as well as biofilm state [22,25].

Meanwhile, due to the increasing development of resistance to quinolones which is associated with the overuse of this antibiotic class, personalized and restricted prescription is considered to be crucial [26]. Interestingly, the *P. gingivalis* isolate with the *gyr*A mutation was isolated from a patient with rheumatoid arthritis. Since quinolones are rarely used in periodontal therapy in Switzerland, this resistance may be associated with the treatment of non-oral bacteria. Certainly, the detection of quinolone-resistant clinical isolates of *P. gingivalis* should raise concerns about the use of antibiotics in the treatment of bacterial-induced oral infections such as periodontitis. Inappropriate use of antibiotics may not only lead to an increase in adverse events and healthcare costs, but might also contribute to the selection of antibiotic-resistant bacteria.

## 4. Materials and Methods

### 4.1. Isolation of Bacteria

All clinical isolates were obtained from subgingival biofilm samples of Swiss periodontitis patients between 1980 and 2017 and stored at −80 °C in the respective strain collections of the oral microbiology diagnostic laboratories in Basel, Bern, and Zurich. The most recent clinical strains originate from two studies approved by the Ethics Committee Bern (#236/2010 and #096/2015). The identification of species changed over time; therefore, the species identity of all the strains was confirmed by using specific 16S rDNA-real-time PCR analysis [27]. In total, 57 *A. actinomycetemcomitans* and 56 *P. gingivalis* strains could be included in the analysis. For resistance testing, these isolates were then cultured on Tryptic Soy agar plates with 5% sheep blood (Oxoid, Thermo Fisher Diagnostics AG, Pratteln, Switzerland), and incubated anaerobically.

### 4.2. Determination of the Minimal Inhibitory Concentration (MIC) and Resistance Testing

MICRONAUT-S Anaerobe MIC plates (Merlin Diagnostica, Bornheim-Hersel, Germany) were used for determining the minimal inhibitory concentrations (MICs) for the following antibiotics:ampicillin (range: 0.063–8 µg/mL), amoxicillin/clavulanic acid (range: 0.5/0.25–64/32 µg/mL), clindamycin (range: 0.063–8 µg/mL), ertapenem (range: 0.125–16 µg/mL), doxycycline (range: 0.125–16 µg/mL), metronidazole (range: 0.25–32 µg/mL), and moxifloxacin (range: 0.063–8 µg/mL). The tests were performed according to the manufacturer’s recommendations. For azithromycin (range: 0.063–8 µg/mL), a microbroth dilution assay was used.

Elevated MICs were confirmed by an agar dilution technique using Wilkins Chalgren agar plates with 5% laked horse blood (Oxoid, Thermo Fisher Diagnostics AG, Pratteln, Switzerland). All the strains were screened for beta-lactamases using nitrocefin discs as described by the manufacturer (Sigma-Aldrich, Buchs, Switzerland). The presence of the *cfx*A, *erm*F, and *tet*Q genes was determined by real-time PCR assays using GoTaq^®^ qPCR Master Mix (Promega AG, Dübendorf, Switzerland) and specific primers [28]. A strain with an elevated resistance against moxifloxacin was forwarded to PCR analysis of the *gyr*A quinolone resistant determining region (QRDR) [13] and subsequent sequencing of the PCR product (Microsynth AG, Balgach, Switzerland).

The MIC results were interpreted using EUCAST breakpoints for anaerobic bacteria in the case of *P. gingivalis*. In the case of *A. actinomycetemcomitans*, the interpretive criteria for the HACEK-group were applied [29]. The distribution of the MIC values is shown by presenting the cumulative MIC values and by calculating the necessary concentrations to inhibit 50% (MIC_50_) and 90% (MIC_90_) of the respective strains.

### 4.3. Statistical Analysis

The descriptive analysis and Chi^2^ test were done using SPSS 24.0 (IBM, Chicago, IL, USA).

## 5. Conclusions

There was no significant increase in the antibiotic resistance among the oral bacterial isolates tested. However, the detection of resistant *P. gingivalis* isolates underlines the need to optimize the antibiotic therapeutic protocols in dentistry.

## Figures and Tables

**Table 1:** Temporal distribution of *A. actinomycetemcomitans* and *P. gingivalis* isolates.

Years	*A. actinomycetemcomitans*	*P. gingivalis*
1980–1989	22	*na*
1990–1999	19	25
2000–2009	8	11
2010–2017	8	20

*na*: no isolates available.

**Table 2:** MIC_50_ and MIC_90_ values (µg/mL) for *A. actinomycetemcomitans* and *P. gingivalis* strains.

Antibiotic	*A. actinomycetemcomitans*		*P. gingivalis*	
	MIC50	MIC90	MIC50	MIC90
Ampicillin	0.5	2	0.125	0.5
Amoxicillin/Clavulanic Acid	0.5/0.25	2/1	0.5/0.25	0.5/0.25
Clindamycin	8	>8	0.0625	0.25
Azithromycin	0.25	1	0.250	0.5
Ertapenem	0.125	0.25	0.125	0.25
Doxycycline	0.5	1	0.25	1
Moxifloxacin	0.0625	0.125	0.0625	0.125
Metronidazole	32	>32	0.25	2
